# Recreational and occupational field exposure to freshwater cyanobacteria – a review of anecdotal and case reports, epidemiological studies and the challenges for epidemiologic assessment

**DOI:** 10.1186/1476-069X-5-6

**Published:** 2006-03-24

**Authors:** Ian Stewart, Penelope M Webb, Philip J Schluter, Glen R Shaw

**Affiliations:** 1 National Research Centre for Environmental Toxicology, University of Queensland, 39 Kessels Road, Coopers Plains, QLD 4108, Australia; 2 School of Population Health, University of Queensland, Herston Road, Herston, QLD 4006, Australia; 3 Cooperative Research Centre for Water Quality and Treatment, PMB 3, Salisbury, SA 5108, Australia; 4 Queensland Institute of Medical Research, Herston Road, Herston, QLD 4006, Australia; 5 Faculty of Health and Environmental Sciences, Auckland University of Technology, Private Bag 92006, Auckland 1020, New Zealand; 6 School of Public Health, Griffith University, University Drive, Meadowbrook, QLD 4131, Australia

## Abstract

Cyanobacteria are common inhabitants of freshwater lakes and reservoirs throughout the world. Under favourable conditions, certain cyanobacteria can dominate the phytoplankton within a waterbody and form nuisance blooms. Case reports and anecdotal references dating from 1949 describe a range of illnesses associated with recreational exposure to cyanobacteria: hay fever-like symptoms, pruritic skin rashes and gastro-intestinal symptoms are most frequently reported. Some papers give convincing descriptions of allergic reactions while others describe more serious acute illnesses, with symptoms such as severe headache, pneumonia, fever, myalgia, vertigo and blistering in the mouth. A coroner in the United States found that a teenage boy died as a result of accidentally ingesting a neurotoxic cyanotoxin from a golf course pond. This death is the first recorded human fatality attributed to recreational exposure to cyanobacteria, although uncertainties surround the forensic identification of the suspected cyanotoxin in this case.

We systematically reviewed the literature on recreational exposure to freshwater cyanobacteria. Epidemiological data are limited, with six studies conducted since 1990. Statistically significant increases in symptoms were reported in individuals exposed to cyanobacteria compared to unexposed counterparts in two Australian cohort studies, though minor morbidity appeared to be the main finding. The four other small studies (three from the UK, one Australian) did not report any significant association. However, the potential for serious injury or death remains, as freshwater cyanobacteria under bloom conditions are capable of producing potent toxins that cause specific and severe dysfunction to hepatic or central nervous systems. The exposure route for these toxins is oral, from ingestion of recreational water, and possibly by inhalation.

A range of freshwater microbial agents may cause acute conditions that present with features that resemble illnesses attributed to contact with cyanobacteria and, conversely, acute illness resulting from exposure to cyanobacteria or cyanotoxins in recreational waters could be misdiagnosed. Accurately assessing exposure to cyanobacteria in recreational waters is difficult and unreliable at present, as specific biomarkers are unavailable. However, diagnosis of cyanobacteria-related illness should be considered for individuals presenting with acute illness following freshwater contact if a description is given of a waterbody visibly affected by planktonic mass development.

## Introduction

Cyanobacteria are a diverse group of prokaryotes that occupy a broad range of ecological niches by virtue of their age, having first appeared some 2.5 billion years ago, and specialisation. All cyanobacteria are photoautotrophic organisms, yet many can grow heterotrophically, using light for energy and organic compounds as a carbon source [1]. The cyanobacteria are a remarkably widespread and successful group, colonising freshwater, marine and terrestrial ecosystems, including extreme habitats such as Antarctic lakes, salt works and hot springs [2]. Cyanobacteria are common inhabitants of freshwater lakes and reservoirs throughout the world. Under favourable conditions, certain cyanobacteria can dominate the phytoplankton within a waterbody and form nuisance blooms.

Cyanobacteria have come to the attention of public health workers because many freshwater and brackish species can produce a range of potent toxins. This observation was first reported over 120 years ago, when sheep, horses, dogs and pigs were seen to die within hours of drinking from a lake affected by a bloom of the brackish-water cyanobacterium *Nodularia spumigena *[3]. Since then, many reports of livestock and wild animal deaths have appeared in the literature. Such reports have been collated and discussed by several authors [4-10]. Some reports are dramatic in terms of the number of animals affected or the rapid progression of illness and death, with mass deaths of thousands of animals [11], and large animals succumbing within minutes [12]. Laboratory-based toxicological investigations have confirmed that freshwater and brackish cyanobacteria produce several categories of toxin that are (with one exception – the saxitoxins) unique to cyanobacteria. The topic of cyanobacterial toxins has been widely studied, and many excellent texts and reviews are available, e.g. [8-10,13-26]. Details of the principal cyanotoxin groups that are significant from the public health perspective of acute exposure and outcome are summarised in Table 1. Lipopolysaccharides, which are defining structural components of the cell walls of Gram-negative bacteria, are discussed in the accompanying review by Stewart et al [27]. Cyanobacteria are rich sources of bioactive compounds; structurally diverse metabolites with cytotoxic, tumour-promoting and enzyme-inhibiting properties are known and presumably many more await discovery. Some of these metabolites are discussed by Bickel et al[28] and Forchert et al [29].

**Table 1:** Cyanotoxins with public health significance from acute exposures

**Toxin or toxin group**	**Classification by principal target organ systems**	**Toxin-producing genera**	**LD_**50**_(i.p. mouse)**	**References**
Microcystins	Hepatotoxins	*Anabaena, Anabaenopsis, Aphanocapsa, Arthrospira, Hapalosiphon, Microcystis, Nostoc, Oscillatoria, Planktothrix, Snowella, Woronichinia*	25->1000 μg/kg	[10, 19, 26, 125-128]
Nodularins	Hepatotoxins	*Nodularia*	30–60 μg/kg	[8, 26, 129]
Anatoxin-a, homoanatoxin-a	Neurotoxins	*Anabaena, Aphanizomenon, Arthrospira, Cylindrospermum, Microcystis, Oscillatoria, Phormidium, Planktothrix, Raphidiopsis*	200–375 μg/kg	[8, 10, 18, 26, 130-135]
Anatoxin-a(s)	Neurotoxin	*Anabaena*	20–40 μg/kg	[8, 26, 132]
Saxitoxins	Neurotoxins	*Anabaena, Aphanizomenon, Cylindrospermopsis, Lyngbya, Planktothrix*	10–30 μg/kg	[26, 127, 132, 136-140]
Cylindrospermopsin	General cytotoxin (multiple organ systems affected, incl. liver, kidney, gastrointestinal tract, heart, spleen, thymus, skin)	*Anabaena, Aphanizomenon, Cylindrospermopsis, Raphidiopsis, Umezakia*	2.1 mg/kg (24 hours) 200 μg/kg (5–6 days)	[8, 10, 17, 132, 141-145]
Aplysiatoxin, debromoaplysiatoxin	Dermal toxins; probable gastro-intestinal inflammatory toxin	*Lyngbya*	107–117 μg/kg	[146-152]
Lyngbyatoxin A	Possible gastro-intestinal inflammatory toxin	*Lyngbya*	250 μg/kg (?LD_100_)	[153]

A recent report has shown that *β-N*-methylamino-L-alanine (BMAA), a neurotoxic non-protein amino acid associated with an atypical motor neurone disease/Parkinsonism/Alzheimer's-like dementia complex, is produced by a wide variety of cyanobacteria [30]. BMAA is thought to be capable of binding to endogenous proteins, in which form it may function as a "slow toxin", and may be implicated in the aetiology of other long-latency neurodegenerative diseases such as Alzheimer's disease [31]. The public health implications of this cyanobacteria-related neurotoxicity hypothesis have been further discussed [32].

Cyanobacterial poisoning of humans has occurred through known and suspected exposure to cyanotoxin-contaminated drinking water supplies [33] and reviewed in: [8-10,34]; confirmed and suspected exposure to contaminated dialysate in patients undergoing haemodialysis [35-39]; and through recreational and occupational contact. This review will concentrate on the latter exposures.

### Rationale and search criteria

All references that could be found in the medical and scientific literature, including conference proceedings, which describe specific incidents involving human illness and exposure to freshwater cyanobacteria in recreational or in-field occupational settings are summarised in Additional File 1. The following citation sources were not examined for this exercise:

• Reports of cyanobacteria-associated illness from recreational exposures to marine or estuarine waters.

• Publications written in languages other than English – with the exception of three papers which we were opportunistically able to have translated [40-42].

• Newspaper reports – with three exceptions: two reports describe the first human fatality to be attributed to recreational contact with cyanobacteria [43,44]. At the time this review was submitted, these were apparently the only published references to describe the events surrounding this tragedy, so were included here because of their importance. The cyanobacteria research community awaits publication of a comprehensive case report in the scientific or medical literature. Another news article supplements a cursory description in an academic journal (though not a health-related journal) of cyanobacteria-associated illnesses; both the news report and the scientific publication appear to describe the same incident, with more detail provided by the journalist [45,46]. There are undoubtedly many more publications in the news media that report suspected cyanobacteria-related human and animal morbidity and mortality: for example Duggan [47] and Ruff [48] reported on cyanobacteria blooms in Nebraska lakes that were associated with two dog deaths and more than 40 complaints of acute eye, upper respiratory, gastrointestinal and skin symptoms.

Anecdotal and case reports presented in this review were identified by the following search strategy:

1. PubMed and Web of Science electronic databases were searched with the MeSH and textword string "(cyanobacter* AND disease outbreaks) OR (cyanobacter* AND environmental exposure) OR (cyanobacter* AND recreation*) OR (cyanobacter* AND epidemiology)".

2. Titles and abstracts (when available electronically) were perused to determine suitability for inclusion.

3. Bibliographies of identified primary papers and related review articles were reviewed to search for references not identified by electronic sources.

4. Publications and other sources identified and forwarded by experts working in this field were included.

The most recent update of the aforementioned electronic searches, conducted in June 2005, gave 257 citations, of which 244 were English-language publications and 13 were non-English-language papers. Of these 13 reports, three (two reviews and one primary article) were identified from abstracts and/or article titles as worth perusing for the presence of information about health-related events associated with recreational exposure to cyanobacteria [41,49,50]. One of these papers (the primary article, in Dutch) and another German review paper we found with a different search strategy were translated for us, but there were no previously unreported references in those papers to specific illness events that were attributed to contact with cyanobacteria [41,42]. Therefore it does not appear that there is a significantly large body of unexplored literature written in languages other than English that could contribute to this review. We also corresponded with an author of a publication in Finnish that we were unable to have translated; this paper discussed cyanobacteria-related illness in saunas [51]. The findings of that work were presented at an international conference, from which an English-language abstract was published. The authors reported that 18 subjects (38% of those questioned) were likely to have experienced cyanobacteria-related symptoms [52].

### Recreational and in-field occupational exposure to cyanobacteria: anecdotal and case reports

Case reports and anecdotal references presented in Additional File 1 date from 1949 [53], and describe a range of illnesses associated with recreational exposure to cyanobacteria: hay fever-like symptoms, pruritic skin rashes and gastro-intestinal symptoms are most frequently reported. Some papers give convincing descriptions of allergic responses to cyanobacteria [53,54]. Others describe more serious acute illnesses, with symptoms such as severe headache, pneumonia, fever, myalgia, vertigo and blistering in the mouth [6,55-57]. The first and so far only description of a fatality attributed to recreational exposure to cyanotoxins appeared in news reports recently [43,44]. A U.S. coroner concluded that a teenage boy died as a result of ingesting anatoxin-a-producing cyanobacteria from a golf course pond, although there was an unusual sequence of events preceding the death insofar as the time period between exposure and death (some 48 hours) does not tally with the known mechanisms of toxicity of anatoxin-a, which initiates pathological signs and death in laboratory animals within minutes of dosing by either oral or parenteral routes [58-60]. Animals exposed to anatoxin-a-producing cyanobacteria in the field succumb within minutes to a few hours, depending on the species, the amount of toxin consumed, and prior food intake [61]. However, Rogers et al [60] demonstrated delayed mortality in toad embryos – over 6–13 days post-exposure – to anatoxin-a. Recent reports in the scientific literature also add to the uncertainty in the case of the teenager's death, with suggestions that the preliminary mass spectrometric identification of anatoxin-a in the forensic samples may not be reliable [62-64].

The principal public health concerns regarding recreational exposures relate to the potential, presumably a now-realised potential if the aforementioned fatality is indeed attributable to cyanotoxin poisoning, for exposure to hazardous levels of cyanotoxins in untreated waters. Routes of exposure are through direct contact with skin and mucous membranes, via inhalation, and by ingestion, either accidental or deliberate.

### Discussion of anecdotal and case reports

Some reports listed in Additional File 1 present scant information relevant to this topic, with little or no detail beyond location and the kind of illness reported [65,66]. On the other end of the scale are examples of thorough, considered case reports, describing relevant medical history and diagnostic investigations [53,54]. One reason for the dearth of detail may be that non-specific, mild and self-limiting illnesses do not merit much discussion, however, some references to more serious illnesses leave a great deal unanswered, for example the 12 year-old boy who reportedly lapsed into unconsciousness for a six-hour period, and developed pneumonia, myalgia and arthralgia [67]. It would have been very interesting to know whether or not this boy had any predisposing medical conditions (e.g. diabetes, epilepsy) that might have explained the loss of consciousness, whether any medical attention was sought, and, if so, the details of his disease progression.

The observation that repeated water contact in a particular lake preceded a skin eruption on a six year-old girl, while other bathers appeared unaffected, helped support a diagnosis of hypersensitivity in that case [54]. One of the few reports of mass effects, with 20–30 children suffering conjunctival and upper respiratory symptoms during a school aquatic event, is tempered by the observation that that number represented about 25% of those exposed [68]. So hypersensitivity reactions affecting a sub-set of allergy-prone children may also be an explanation for the latter outbreak, although this speculation – in the absence of any other reported investigations – is solely based on that estimate of 25% of those exposed experiencing symptoms.

Those reports that have indicated symptom onset time suggest that responses can be rapid, with some urticarial and hay fever-like symptoms commencing while subjects are still in the water [53,68]. While a disparate range of signs and symptoms are listed, several reports describe a collective group of symptoms resembling immediate or Type-I hypersensitivity reactions. Immediate hypersensitivity reactions are commonly associated with atopy, which is the familial tendency to react to naturally occurring antigens, mostly proteins, through an IgE-mediated process. Atopy frequently manifests as a spectrum of diseases, e.g. seasonal rhinitis, conjunctivitis, asthma and urticaria. Different atopic illnesses often affect the same individual. A fundamental feature of Type-I hypersensitivity reactions is the rapid onset of symptoms – normally seconds to minutes – following exposure to antigens [69-73].

Some serious though apparently self-limiting gastro-intestinal illnesses have been reported after contact with cyanobacteria in recreational waters, presumably through ingestion of affected water. Dillenberg & Dehnel [55] describe how an adult male inadvertently swallowed lake water affected by a bloom of *Microcystis *sp. and *Anabaena circinalis*. After some three hours he developed cramping abdominal pain and nausea, which progressed to painful diarrhoea followed by a fever of 38.9°C, severe headache, lassitude, myalgia and arthralgia. Such illnesses are worrying, considering the two boys that were sickened – one of whom subsequently died – after possible exposure to cyanobacteria in a golf course pond suffered acute and severe gastro-intestinal illnesses [43].

Occupational exposures were included in this review, although some caution should be exercised when comparing occupational and recreational exposures. Waters that are obviously discoloured or visibly affected by cyanobacteria scums may be of interest to aquatic field workers who are keen and/or obliged to collect samples. The two incidents involving British soldiers and sea cadets conducting canoe capsizing activities, presumably under orders from their supervising officers, occurred in waters that were reportedly subject to a "heavy bloom of *Microcystis *spp" [74] and a "scum of *Oscillatoria*..." [23]. Waters that are obviously suffering a loss of visual amenity may be shunned by many recreational users, although avoidance behaviour in such circumstances cannot be taken for granted [75].

The other reports that are of particular interest are those grouped under "cold & flu-like symptoms". Several publications describe individuals presenting with a flu-like illness, with signs and symptoms including fever, headache, lassitude, arthralgia, myalgia, sore throat, cough, diarrhoea and vomiting. A proposed explanation for this constellation of symptoms is that of a coordinated, cytokine-mediated innate immune response. Fever and malaise are events that are directed by endogenous mediators; for further discussion see [75]. This spectrum of signs and symptoms also mimics those reported in volunteer studies of intravenous Gram-negative bacterial LPS injection [76-79]. Mammalian responses to LPS are mediated by inflammatory cytokines (see accompanying review by Stewart et al [27]). The early signs and symptoms of influenza infection (fever, myalgia, fatigue, drowsiness, rhinorrhoea, sore throat, headache) are mediated by pro-inflammatory cytokines, particularly IFN-α and IL-6 [80-83]. Flu-like reactions to immunostimulant drugs are sometimes referred to as "acute cytokine syndromes" [84], and the flu-like syndrome of fever, rigors, tachycardia, malaise, headache, arthralgia and myalgia that accompanies interferon pharmacotherapy is thought to be due to the release of eicosanoids, IL-1 and TNF-α [85].

### Epidemiology of recreational exposure to cyanobacteria

Six epidemiological studies of recreational exposure to cyanobacteria were identified with the search strategy discussed previously: three analytical cross-sectional studies from the U.K. using similar survey instruments [86-88], a small case-control study from Australia [89], and two larger prospective cohort studies, also from Australia [90,91]. Table 2 lists the pertinent findings of these studies, which are discussed in detail below.

**Table 2:** Summary of epidemiological studies investigating recreational exposure to cyanobacteria

**Country; year study conducted; study author/s; reference**	**Study design**	**Main outcomes reported**	**Study size (***n***)**	**Odds ratio (95% confidence interval)**
UK, 1990 Philipp [86]	Cross-sectional	No statistically significant findings	246	
UK, 1990 Philipp & Bates [87]	Cross-sectional	No statistically significant findings	363	
UK, 1990 Philipp et al [88]	Cross-sectional	No statistically significant findings	246	
Australia, 1992 El Saadi et al [89]	Case-control	No statistically significant findings	Approx. 48 (subjects reporting recreational exposure)	
Australia, 1995 Pilotto et al [90]	Prospective cohort	Increased symptoms at 7 days following exposure to more than 5,000 cyanobacterial cells/mL for >1 hour *vs *non-bathers	852 (total) 338 (no prior exposure or symptoms)	1.3 (0.7–2.6) 3.4 (1.1–10.8)
Australia & USA, 1999–2002 Stewart et al [91]	Prospective cohort	Increased reporting of mild respiratory symptoms and any symptom at 3 days following exposure to cyanobacteria cell surface area >12 mm^2^/mL *vs *<2.4 mm^2^/mL	1,331 (total) 1,137 (no prior symptoms) 1,149 (no prior respiratory symptoms)	1.7 (1.0–2.9) (any symptom) 2.1 (1.1–4.0) (respiratory symptoms)

The three cross-sectional studies were conducted by Philipp and co-workers [86-88]. Questionnaires were distributed to recreational users of six inland waterbodies, five of which experienced cyanobacteria blooms during 1990. The questionnaires elicited information on exposure to study waters and the presence of specific symptoms in a defined period prior to receiving the form. This period ranged from 14 days [87] to four weeks [88]. One questionnaire asked about exposure to the study water on a weekend when a bloom occurred some 21/2 weeks previously [86]. Recreational interest groups were used to target likely users of the waterbodies; questionnaires were mailed to members of sailing and angling clubs. Site authorities distributed questionnaires at one study lake [88]. The results of these three studies were similar: mostly minor morbidity was reported, with similar disease patterns across sites.

The theoretical advantages of this study type are that it is reasonably cost-effective, and in this context – recreational exposure to cyanobacteria – it can be conducted opportunistically to take advantage of any sudden-onset cyanobacteria blooms. Disadvantages relate to the difficulty in establishing that exposure occurred before the outcome [92,93]. The studies conducted by Philipp and his team [86-88] were examples of analytical cross-sectional studies, in that unexposed individuals served as controls for statistical comparison of illness reporting.

A case-control study of illness rates was conducted after an extensive *Anabaena circinalis*-dominant bloom along South Australia's Murray River in the summer of 1991–1992 [89]. Patients presenting with gastro-intestinal (G-I) or dermatological complaints comprised the case group; the patient presenting after each case was identified served as the control group. Exposure was determined by identifying each subject's principal source of water for drinking, domestic use (bathing, dishwashing) and recreation during the week prior to consultation. Recreational exposure was categorised as no contact, direct exposure to river water, or other exposure, e.g. farm dams or treated water in swimming pools. The study found a significantly increased risk of G-I symptoms for those drinking chlorinated river water, and an increased risk of G-I and cutaneous symptoms in those using untreated river water for domestic purposes. There was a statistically non-significant increase in the relative odds of developing G-I or skin symptoms amongst those with recreational exposure to river water, but that risk was lower than for those exposed to other sources of recreational water (tank, farm dam or another location). The number of subjects was small for the recreational exposure component of the study, with only some 50 subjects (16% of the study group) reporting any recreational exposure during the study period [89].

The advantages of a case-control design for investigating recreational exposure to cyanobacteria are that studies can be conducted opportunistically in response to the development of cyanobacteria blooms, and they are very useful for investigating infrequent outcomes. The study of El Saadi et al [89] has another advantage over other epidemiological studies into recreational exposure to cyanobacteria in that medical practitioners ascertained outcome data, as opposed to self-reporting of symptoms. General disadvantages of the case-control design principally relate to the problem of recall bias, where individuals with the disease of interest tend to overestimate relevant past exposures [92,93]. Because the outcome has already occurred when exposure is measured, people with disease may systematically overestimate (or underestimate) their exposure compared to disease-free controls, leading to falsely elevated (or reduced) measures of risk associated with exposure. Another major issue with case-control studies is the difficulty of identifying an appropriate control group – i.e. people who would have been identified as cases if they had the disease of interest.

Recall bias may not be so much of a problem for investigating acute illnesses following recreational exposure to cyanobacteria, where a fairly short time lag between exposure and symptom onset can be anticipated, especially if recreational exposure is determined by a yes/no response. The main problem with a case-control study in this context will be in actually identifying cases. A case-control design would not be suitable for investigating outcomes from exposure to a cyanobacteria bloom in a lake adjacent to a city, as most recreational users who do develop symptoms would presumably seek medical attention after they return home, i.e. from one of a large number of medical practitioners. El Saadi et al [89] alluded to the difficulty of gaining the cooperation of medical practitioners, as they approached practices in 11 towns along the Murray River, yet those in three towns presumably refused to participate in their study. The diffuse spread of cases from point sources of exposure (a cyanobacteria-affected waterbody) across a large town or city would make a case-control study practically unworkable. A case-control study would also be unsuitable for recruiting subjects who did not seek medical attention for symptoms occurring after exposure. However, a well-designed case-control study would be valuable if geographical location is a primary consideration. This would require enlisting the cooperation of medical practitioners in small townships near to cyanobacteria-affected recreational waters that are sufficiently remote from larger urban centres to allow recruitment of local residents and tourists who will camp nearby.

The studies by Pilotto et al [90] and Stewart et al [91] were prospective cohort studies. Pilotto et al [90] recruited individuals at five recreational waterbodies in three Australian states. Cyanobacteria blooms were anticipated at these sites, based on occurrences in previous years. Individuals were approached and invited to participate in the study. Participants completed a face-to-face interview to determine health status and recreational water activities; two telephone follow-up interviews were conducted at two and seven days following the day of recruitment into the study. Individuals who did not have water contact on the recruitment day served as the control group. No significant differences in symptom occurrence were reported at the 2^nd ^day follow-up, but the authors concluded there was a significant increase in symptoms at 7 days, after excluding subjects with symptoms or previous recent recreational water exposure. The cohort size from which these significant results were drawn was rather small, with 93 exposed subjects, and 43 unexposed controls. Pilotto et al [90] interpreted the increased symptom reporting at 7 days but not 2 days following exposure as possibly due to delayed allergic responses, although so-called "late phase" allergic and asthmatic responses tend to occur some 4–24 hours after allergen exposure [69,94,95].

Stewart et al [91] also conducted a cohort study of recreational exposure to cyanobacteria. 1,331 subjects were recruited from 19 recreational waterbodies in eastern Australia and central and northeast Florida; subjects completed a self-administered questionnaire to determine recreational activity, recent illness and history of any relevant chronic diseases such as asthma, hay fever and eczema. A single follow-up telephone interview was conducted after three days post-exposure. Reference subjects were recruited at recreational waters unaffected by cyanobacteria; exposure categories (low, intermediate, high) were allocated to study subjects on the basis of cyanobacteria levels measured in study water samples collected on the day they were recruited into the study. Statistically significant increased reporting of respiratory symptoms and a pooled "any symptom" category occurred amongst subjects exposed to high levels of cyanobacteria, although symptoms were predominantly rated as mild by study subjects. A similar but non-significant relationship was also seen for reporting of skin, ear and fever symptom groups.

The studies of Pilotto et al [90] and Stewart et al [91] are both examples of a prospective cohort design, where study subjects have their exposure status determined, and are then followed forward in time to observe the development of disease. For these investigations into recreational exposure to cyanobacteria, exposure status was determined by collecting water samples on the day subjects were recruited into the study; cyanobacteria were identified and enumerated and the resultant cell counts or biomass estimates formed the basis of exposure at any given site on a particular day. One of the problems with this approach is that cyanobacteria blooms are dynamic and can change rapidly. Unless the presence of significant cyanobacterial biomass can be predicted with some degree of certainty, a prospective cohort design can result in wasted effort if the water samples reveal lower than anticipated levels of cyanobacteria. This problem undoubtedly occurred in some instances during the study conducted by Stewart et al [91]. One possible approach to dealing with this would be to conduct a historical cohort study, where a cohort of subjects is identified after some have experienced the outcome of interest and relevant exposure information is obtained from historical records (i.e. as in a prospective cohort study the exposure information was recorded *before *any outcomes occurred).

Whether a cohort study is conducted prospectively or retrospectively, the basic study design is identical – exposed and unexposed groups are compared with respect to disease outcome [93]. General advantages of a cohort design are the ability to determine disease onset (the exposure precedes the disease), and the study of exposures in natural settings [92]. General disadvantages relate to confounding, which refers to differences in the distribution of risk factors other than the exposure of interest between exposed and unexposed groups. Cohort studies can be expensive and resource intensive [92].

Further discussion of some common epidemiological study designs that may be useful for investigating the topic of recreational exposure to aquatic cyanobacteria, with particular emphasis on the relative advantages and disadvantages of experimental epidemiology (randomised trials) is presented by Stewart [75].

### Cyanobacteria and water-related disease: some complicating factors

Other explanations for disease need to be considered by both clinicians and epidemiologists in their respective endeavours. Epidemiological studies usually aim to identify and adjust for confounding variables such as smoking and age of study participants. The following sections will discuss some freshwater-related risk factors, mostly microbial, that may confound epidemiological studies and complicate clinical diagnoses of cyanobacteria-related illness linked to recreational exposures. The final section of this review will discuss the possibility of misdiagnosis from the opposite direction: a water-borne disease outbreak in Finland that was subject to epidemiological scrutiny, but cyanobacterial exotoxin contamination of reticulated supplies was apparently not considered at the time.

### Freshwater-related dermatoses

• **Avian cercariae: **avian cercariae are schistosome larvae for which humans are an accidental host. Pruritus and macules are the initial signs and symptoms; sometimes a diffuse erythema and urticaria can develop and last for several hours [96-99]. Fever, nausea and vomiting can also accompany severely affected cases [97,100]. The clinical presentation of cercarial dermatitis can be difficult to delineate from the picture of cyanobacterial dermatitis.

• **Gram-negative bacteria: ***Aeromonas hydrophila *and *Chromobacterium violaceum *are abundant in freshwater habitats. Both usually cause infection through a pre-existing skin wound, though the clinical presentations in each case do not match of any of the reports listed in Additional File 1. *A. hydrophila *causes cellulitis and a purulent discharge; aspiration of water can cause pneumonia and septicaemia. *C. violaceum *infections present with various cutaneous signs that are secondary to systemic disease, including sepsis [101]. *Vibrio vulnificus *has reportedly caused soft tissue infection after contact in brackish inland waters, though most cases are associated with estuarine contact [102]. *Pseudomonas aeruginosa *is widely-distributed in natural and artificial aquatic environments. Cutaneous infection presents as an erythematous or urticarial rash some 18–24 hours after water contact and progresses to a follicular dermatitis. Fever and pruritus are uncommon. Most reports of pseudomonal dermatitis are related to spa pool or hot-tub exposures [102,103]. *P. aeruginosa *in recreational waters is a common cause of otitis externa, presenting as a purulent discharge [102]. Diagnostic criteria include culturing the organism from skin or ear swabs; the incubation period would also help to distinguish *P. aeruginosa *infection from cyanobacteria-related dermatoses.

• **Non-allergic urticaria: **physical stimuli such as heat, cold and exercise can induce itching and hives in susceptible individuals [99,104].

### Gastro-intestinal illness

• **Shigellosis: **Shigella outbreaks are the most commonly reported cause of disease associated with untreated inland recreational water in the USA, with 16 events affecting almost 1,300 people between 1985 and 1994 [102]. The incubation period is typically 2–3 days, with an upper limit of about 7 days. Illness severity is strain-dependent, with most *S. sonnei *infections being mild and self-limiting, and *S. dysenteriae *type 1 associated with severe diarrhoea which may progress to a life-threatening illness [102].

• ***Escherichia coli*: ***E. coli *are markers of faecal pollution in recreational waters. Disease outbreaks traced to enterohaemorrhagic *E. coli *0157 have been reported from recreational water exposures [102,105].

• **Norwalk-like viruses: **Various transmission routes, including recreational water outbreaks have been documented [105].

### Other microbial pathogens

• ***Naegleria fowleri*: ***N. fowleri *is a free-living thermotolerant amoeba found in warm or thermally polluted waters. It is the causative organism of primary amoebic meningoencephalitis, a fulminating, typically fatal illness. The entry route is via the nasal mucosa; fit, immunocompetent children and young adults with a recent history of freshwater recreational activity are those most commonly affected. The causative organism and diagnosis are usually confirmed at autopsy. Several reviews are available, e.g. [106-113].

• **Viruses: **Pharyngo-conjunctival fever outbreaks associated with non-enteric adenoviruses in recreational waters have been reported [105].

• **Legionella: ***Legionella *infections have been associated with recreational water contact [105].

### Possible under-diagnosis of cyanobacteria-related illness

The examples given above highlight some of the differential diagnoses that need to be worked through when considering possible cases of cyanobacteria-related illness from recreational exposures. Competent history-taking and diagnostic microbiology support will correctly diagnose many such cases. Competent history-taking and clinical diagnostic support also operated in several of the case reports listed in Additional File 1, with the early dermatological testing and microscopic examination of stool and vomitus samples lending strong support to the suspicion of cyanobacteria-related morbidity.

Misdiagnosis of cyanobacteria-related disease may occur in both directions. In 1978, nearly half the population of an industrial town in Finland were affected by a flu-like illness, with symptoms of fever, fatigue, cough, dyspnoea and myalgia. Symptoms occurred some 3–6 hours after taking a bath, shower or sauna and persisted for 8–16 hours. The outbreak lasted for some four months. This epidemic was investigated on several fronts, and provocation testing demonstrated an obvious link to the reticulated water supply. Tap water was cultured in a range of organic media for fungal and bacterial pathogens. No definitive pathogen was identified to explain the epidemic, yet in three published reports the authors describe how the shallow lake that was the town water source had taken on a distinct opaque blue-green appearance, had a musty smell, and the sand filtration system was covered by a mat of cyanobacteria. This change occurred in the same month (August, i.e. late summer) that the epidemic began. Analysis of crude lake water in the third month after the onset of the epidemic showed high coliform counts, *Aspergillus fumigatus *and unspecified blue-green algae. Investigations centred on identifying antibodies to mesophilic actinomycetes, which the authors [114] note were not pathogenic, whereas aquatic cyanobacteria were known at the time to be toxic. The health workers investigating the outbreak apparently did not consider the possibility of a cyanobacterial exotoxin breakthrough into the reticulated supply [114-118]. The epidemiological report of Aro et al [115] came closest to suggesting that cyanobacteria may have been involved, suggesting that "towards the end of summer....the microorganisms in the lake multiply rapidly and produce some toxic substance or allergen", and reported that cyanobacterial endotoxin concentration in lake and tap water was high. This incident appears to have been retrospectively attributed to the presence of cyanobacterial endotoxins (i.e. LPS) in the reticulated supply [119]. A similar outbreak occurred almost three years earlier in a Swedish town, though with a much smaller proportion of cases identified. Cyanobacteria were known to affect the town's raw water supply, and the investigators did consider the possibility that cyanotoxins may have been responsible for the outbreak [120], though the analytical technique used by investigators at the time – gas chromatography – would have failed to detect the presence of cyanobacterial exotoxins in the post-treatment water supply. While no conclusions can be made about events that occurred over 25 years ago, from the descriptions of the outbreaks and the raw water supplies, most cyanobacterial toxicologists would rate cyanotoxin exposure with a high index of suspicion.

A similar outbreak occurred more recently in Homa Bay, Kenya, in 1998. Apparently associated with a mass development of cyanobacteria in Lake Victoria, an epidemic of fever, malaise, dizziness and upper respiratory symptoms was related to hot water bathing. Symptoms lasted 12–24 hours, and returned when a shower or bath was taken again. This outbreak was reported in a conference abstract; the authors suggested cyanobacterial endotoxins were responsible, though it is not stated whether any investigation of cyanobacterial exotoxins was conducted [121].

## Conclusion

The true incidence of acute cyanobacteria-associated illness from recreational exposure is unknown, as many outcomes are likely to be mild and self-limiting, so medical attention is not sought. With a long-standing knowledge gap amongst primary healthcare providers, non-specific signs and symptoms caused by cyanobacterial products are likely to be under-diagnosed [8]. Codd [122] stated:

"Evidence linking human illnesses with cyanobacterial cells and toxins is open to criticism because of shortfalls in early detailed case definitions, because diagnoses were made by exclusion, and because identification and quantification of cyanobacterial toxins in health incidents have, until recently, been lacking."

The collation of anecdotal and case reports of illness associated with recreational exposure to cyanobacteria in Additional File 1 will hopefully highlight some of the knowledge gaps. Particular attention should be given to determining the onset and duration of individual symptoms in future case reporting, as well as detailing the presence or absence of any predisposing medical conditions.

A recent initiative of UNESCO's International Hydrology Programme has been to establish CyanoNet, which is a "Global network for the hazard management of cyanobacterial blooms and toxins in water resources". The CyanoNet website will carry information on various associated topics, including "Reported incidents of adverse health effects including case studies" and "Surveys and epidemiological studies investigating associations between cyanobacterial populations, cyanotoxins and health" [123].

The most important advances in understanding the health impacts of cyanobacteria have come from the discipline of toxicology. The major toxins have been extensively studied and characterised, and while there is still much to be discovered in the field of cyanobacterial toxicology, significant advances in the future will be made at the interface of toxicology and epidemiology. Molecular epidemiology techniques using yet-to-be discovered biomarkers of exposure, susceptibility and outcome will refine knowledge of the risks associated with various acute and chronic exposures to cyanotoxins. The collaborative skills that epidemiologists and toxicologists can bring to this endeavour were viewed with a mildly jaundiced eye by Paddle [124], whose chapter on epidemiology for toxicologists is an excellent general primer:

"The total evidence about the risk to humans...will consist of the toxicologist's precise, experimental data about the wrong species at the wrong exposure, and the epidemiologist's imprecise, observational data about the right species at the right exposure."

In conclusion, anecdotal and case reports of variable reliability have suggested a range of symptoms are associated with exposure to cyanobacteria in recreational or occupational settings. Some reports of cutaneous reactions are strongly suggestive of allergic reactions, and symptoms such as rhinitis, conjunctivitis, asthma and urticaria also hint at immediate hypersensitivity responses. Flu-like illnesses involving a constellation of symptoms including fever, malaise, myalgia, arthralgia, severe headache, cough and sore throat are, in our opinion, explained by a cascade action of pro-inflammatory cytokines. If correct, this implies that some cyanobacterial products possess ligands that induce innate immune responses, and such responses may need to be considered in terms of their potential to direct pathological changes in the liver and other organ systems.

The epidemiology of recreational exposure to cyanobacteria is incomplete at present. All common epidemiological approaches have their own inherent advantages and disadvantages; identification of biomarkers for exposure, susceptibility and outcome in the future should lead to a significantly improved perception of the risks of bathing in cyanobacteria-affected waters.

## Abbreviations

BMAA *β-N*-methylamino-L-alanine

G-I gastro-intestinal

GP General Practitioner (aka Family Physician)

IFN interferon

IgE immunoglobulin-E

IL interleukin

i.p. intra-peritoneal

LD_50 _lethal dose for 50% of test animals

LPS lipopolysaccharide(s)

TNF-α tumour necrosis factor-alpha

UNESCO United Nations Educational, Scientific and Cultural Organization

## Competing interests

The author(s) declare that they have no competing interests.

## Authors' contributions

IS conducted the review; PMW, PJS and GRS supervised the work and contributed to redrafting the paper. All authors read and endorsed the final manuscript.

## Supplementary Material

Additional File 1 Anecdotal and case reports of human morbidity and mortality attributed to recreational or occupational field exposure to freshwater cyanobacteria. Click here for file
