# Supplementary material for: Recreational and occupational field exposure to freshwater cyanobacteria – a review of anecdotal and case reports, epidemiological studies and the challenges for epidemiologic assessment
Source: Environ Health. 2006 Mar 24;5:6. doi: 10.1186/1476-069X-5-6 (PMC1513208; doi:10.1186/1476-069X-5-6)
Supplement: Additional File 1 — Anecdotal and case reports of human morbidity and mortality attributed to recreational or occupational field exposure to freshwater cyanobacteria. [file 1476-069X-5-6-S1.pdf]

Anecdotal and case reports of human morbidity and mortality attributed to recreational or occupational field exposure to freshwater cyanobacteria

Year	Season	Location	Number affected	Estimates of number unaffected	Age	Water activity	Signs & symptoms	Dominant plankton	Time of onset after exposure	Symptom duration	Diagnostic criteria	Predisposing conditions	Notes	References
**Hayfever-like symptoms (conjunctivitis, rhinitis, sneezing)**														
1934	Late summer	Muskego Lake, Waukesha County, WI, USA	Report of single subject, male	N/S	57	Swimming	Itching of eyes, nasal congestion	"weedy lake"	3 hours	<48 hours		History of frequent sinus infections		[53]
1935	Late summer	Muskego Lake, WI, USA	Same subject	N/S		Swimming	Same symptoms, + mild asthma		As above	As above				[53]
1936-1946	Late summer	North Lake, Waukesha County, WI, USA	Same subject	N/S		Swimming	Nasal discharge and congestion, conjunctivitis, mild asthma	Oscillatoreaceae (sample taken in late summer, 1944)	N/S	N/S	Surface scum extracts gave immediate skin reactions. Cutaneous injection of 0.03mL of 1:1,000 dilution resulted in mild asthma within 20 mins. Control subjects did not react to scum extracts		Subject swam in North Lake during summer months over the ten-year period, without incident until mid-August each year, when swimming was followed by symptom onset. Desensitisation injections over four years were successful	[53]
1979	Late summer	Lake Wallenpaupack, PA, USA	5 (family group)	N/S	N/S (parents + son + daughter + friend)	Swimming	"Severe" hayfever-like symptoms (sneezing, nasal discharge, eye irritation): 3/5 earache: 2/5	N/S. Lake developed a distinct green colour several days prior to incident. Three weeks later: heavy bloom of *Anabaena*	Hayfever symptoms: during exposure; earache: several hours after exposure	N/S		N/S		[68]
1979	Late summer	Lake Wallenpaupack, PA, USA	20-30	60-90 (those affected comprised approx 25% of aquatic event participants)	N/S (high school students)	Participating in school aquatic event	Eye irritation, sore throat, earache, sneezing, nasal discharge, swollen lips	N/S. see above	During exposure or within 2-3 hours	Within 2-3 days<br><br>*Subsequent exposure reportedly caused re-occurrence of symptoms		N/S	Affected individuals were members of a high school summer aquatic program. The event was cancelled because of these illnesses	[68]
N/S	N/S	N/S	Single male	N/S	N/S (adult)	Swimming	Hayfever-like symptoms	Bloom of *Microcystis*	N/S	N/S		N/S		[154]

Year	Season	Location	Number affected	Estimates of number unaffected	Age	Water activity	Signs & symptoms	Dominant plankton	Time of onset after exposure	Symptom duration	Diagnostic criteria	Predisposing conditions	Notes	References
**Cutaneous symptoms**														
1949-1952	Summer	Lake Carey, PA, USA	Report of single subject, female	Reported skin rash "never appeared in other bathers swimming in the same water"	6 (condition first seen at age 3 years)	Swimming	Seasonal erythematous papulo-vesicular rash, occasionally progressing to oozing and crusting. Rash limited to face, neck, shoulders, upper chest and extremities; rash never seen on areas covered by swimsuit	*Anabaena*	1-2 hours	< 2 weeks (if no further contact with lake water)	Skin patch testing: strong positives to *Anabaena* filtered from lake water, chloroform extract of same, and phycocyanin extract	None (apart from reported condition)	Rash never seen after swimming in artificial pools or ocean. Only seen after water exposure at Lake Carey, and once after bathing in a Canadian lake	[54]
Late 1940s & mid 1950s	Late summer	Lake Ringsjön, Scania, Sweden	"several" members of a family	N/S	N/S ("young girl" + siblings)	Swimming	Pruritic skin rashes	*Gloeotrichia echinulata*	N/S	N/S				[155, 156]
1977	Summer	Mingechaur Reservoir, Azerbaijan	7	13	N/S	Swimming	Slightly raised, erythematous spots, 2-6mm diameter; seen on skin bordering and outside swimsuit	Benthic *Lyngbya kützingii*; also "planktonic blue-green algae colouring the water"	2nd day after swimming	10-12 days				[40]
1985	N/S	UK	N/S	N/S	N/S	Sail-boarding	Skin rashes	N/S ("toxic blooms")	N/S	N/S		N/S		[65]
1989	N/S	Japan (lake)	1	N/S	N/S (adult)	Collecting algal scum *	Rashes	*M. aeruginosa* containing microcystins	N/S	N/S		N/S		[65]
1989	Late summer – early autumn	Bewl Water, Kent, UK	1	N/S	N/S	Fishing	'blotchy' skin on face and hands after handling water on three separate occasions	*Microcystis* sp	Within two hours	Several days		N/S		[65]
1989	N/S	NRA South West Region, UK	N/S ("some NRA staff")	N/S	N/S (adults)	Sampling water *	Tingling sensations on hands	"Potentially toxic cyanobacteria species"	N/S	N/S		N/S		[65]
1989	Early-mid autumn	Welton Water, Yorks, UK	N/S	N/S	N/S	N/S ("water users")	Rashes	*Anabaena* sp	N/S	N/S		N/S		[65]
1991	Late spring – early summer	Darling-Barwon river system, NSW, Australia	N/S	N/S	N/S (adults)	Sampling water*	Skin irritation	*Anabaena* spp	N/S	N/S		N/S		[157]

Year	Season	Location	Number affected	Estimates of number unaffected	Age	Water activity	Signs & symptoms	Dominant plankton	Time of onset after exposure	Symptom duration	Diagnostic criteria	Predisposing conditions	Notes	References
**Cutaneous symptoms**														
2003	Summer	Lake Salajärvi	1	N/S	N/S	Swimming	Rash	*Anabaena lemmermannii* (dominant) + low levels of *M. aeruginosa* and *M. flos-aquae*.	N/S	N/S		N/S	Saxitoxin, microcystins and anatoxin-a products present in water samples	[158]
2003	N/S	Lake Sompanen, Finland	1	N/S	7 yo male	N/S	“extensive” generalised rash	Cyanobacteria visible in water; saxitoxin found in water samples in 2002 & 2004	N/S	N/S		N/S		[158]
**Gastro-intestinal symptoms (nausea, vomiting, diarrhoea, abdominal pain)**														
1959	Summer	One of Katepwa Lakes, SK, Canada	Report of single subject, male	N/S	N/S (adult)	Swimming	Headache, nausea, G-I upset	N/S	During night after swimming	<48 hours.	Stool sample showed “many tiny greenish spheres which resembled in size and morphology the cells of *Microcystis*”. Stool specimen negative for *Salmonella* and *Entamoeba*	N/S	Subject sought medical advice for gastroenteritis, admitted to hospital, given oral chloramphenicol. Recovery within 24 hours of admission	[55]
1959	Summer	Long Lake, SK, Canada	10	N/S	N/S (children)	Swimming	Diarrhoea, vomiting	N/S. Subjects swam in “algae-covered lake water”. Dried *Microcystis* and *Anabaena* scum found later on shore	N/S. Reported to local medical officer with illness on the day after exposure	N/S	Stool specimen from one child contained cells resembling *Anabaena* “in great numbers”		Local farmer reported that two cows died after drinking from the lake 12-16 hours previously, during an algal bloom. A third sick cow recovered after receiving penicillin injections	[55]
N/S	N/S	N/S (lake)	Single subject, male	N/S	4	Fell into lake, swallowed water #	Abdominal pain, nausea, vomiting, diarrhoea, “wooziness”, headache, thirst	N/S	Day of exposure: G-I illness. Next day: wooziness, headache, thirst	N/S	*Aphanizomenon* found in stool and vomitus specimens	N/S		[67]
1961	N/S	N/S	4	N/S	N/S (students)	Swimming	Headache, malaise, diarrhoea	Heavy growth of *Microcystis* and *Anabaena*	N/S	N/S		N/S		[67]

Year	Season	Location	Number affected	Estimates of number unaffected	Age	Water activity	Signs & symptoms	Dominant plankton	Time of onset after exposure	Symptom duration	Diagnostic criteria	Predisposing conditions	Notes	References
**Gastro-intestinal symptoms (nausea, vomiting, diarrhoea, abdominal pain)**														
1989	Early autumn	Rutland Water, Leics, UK	N/S	N/S	2 & 3 year-olds	Playing at edge of scum	Vomiting & diarrhoea	*M. aeruginosa*	N/S	N/S	Microcystin-LR found to be principal cyanobacterial toxin present	N/S	20 sheep and 15 dogs died over approx 3 weeks in late summer – early spring after contact with bloom scum	[6]
1990	N/S	Unnamed lake, Tiel, The Netherlands	N/S	N/S	N/S	Swimming	"G-I complaints"	*Anabaena flos-aquae*	N/S	N/S	Water quality parameters and food-borne pathogen testing negative	N/S	Mouse bioassay and HPLC-UV confirmed presence of anatoxin-a	[159]
2003	Summer	Lake Iso-Kukkanen, Finland	1	N/S	10 yo male	Swimming	Abdominal pains	*A. lemmermannii* sole or dominant cyanobacterium.	N/S	Several hours		N/S	Saxitoxin measured at 13-270 µg/L (depending on methods). Low levels of microcystins found. Anatoxin-a and degradation products reported	[158]
**Progression to fatal illness**														
2002	Mid-summer	Golf course pond, Milwaukee, WI, USA	5	0	17 yo male and 4 friends	"splashing and diving". Two most severely affected boys had their heads under water for varying periods of time #	17-year-old developed nausea, vomiting, progressed to "shock" and "seizure", acute heart failure; death occurred approx. 48 hours after exposure. One teenager who also apparently ingested water developed severe diarrhoea and abdominal pain. The other three youths developed "minor symptoms"	Presumably *A. flos-aquae* containing anatoxin-a, determined from blood and stool samples of the boy that died and the other severely affected youth	N/S	N/S	Analysis of "tissue, blood and other fluid samples" from the two severely affected teenagers. Anatoxin-a found in unspecified sample/s. Autopsy showed "acute heart damage" but no evidence of meningitis or encephalitis. Analyses for pesticides, parasites and other pathogens negative	N/S	The unusual feature of this case is the length of time – 48 hours – between exposure and ingestion of contaminated water and subsequent death. Uncertainty exists regarding the identification of anatoxin-a in tissue samples	[43, 44, 62-64]

Year	Season	Location	Number affected	Estimates of number unaffected	Age	Water activity	Signs & symptoms	Dominant plankton	Time of onset after exposure	Symptom duration	Diagnostic criteria	Predisposing conditions	Notes	References
**Cold & flu-like symptoms (incl. fever, headache, myalgia, arthralgia, sore throat, respiratory and gastro-intestinal)**														
1959	Summer	Echo Lake, SK, Canada	Report of single subject, male	N/S	N/S (adult)	Intending to swim – fell into lake, swallowed an estimated half-pint of water #	Abdominal cramps and pain, nausea, vomiting, painful diarrhoea, fever, severe headache, weakness, pain in limb muscles and joints. Stools and vomitus were slimy and green	N/S. Lake had a visible bloom on day of exposure. *Microcystis* and *Anabaena* bloom 35 days prior to exposure	3 hours: abdominal pain, nausea, vomiting 5 hours: diarrhoea; next morning: fever, headache, myalgia, weakness	Recovering when questioned on 2nd day after exposure	Stool specimen showed "innumerable spheres of *Microcystis* and 2-3 well-preserved curved chains of *A. circinalis* per high-power field"	N/S	During bloom 35 days prior to exposure, an unknown number of dogs and geese died after swimming in the lake. Other dogs sickened after drinking lake water. Also fish kills	[55]
N/S	N/S	N/S (swimming hole)	Single subject, male	N/S	12	Swimming	Fever, loss of consciousness for six hours, dyspnoea, pneumonia, myalgia, arthralgia	Abundant *M. aeruginosa*	N/S (onset reported as sudden, with subsequent myalgia and arthralgia)	N/S	Stool sample: *Aeromonas* (Gram-negative bacteria), *Spirogyra* and *Mougeotia* (both green algae)	N/S		[67]
1979	Late summer	Lake Wallenpaupack, PA, USA	15	N/S	N/S	Swimming	Vomiting, nausea, diarrhoea, eye irritation, sore throat, fever, earache	N/S. See above.	Symptoms occurred "a short time after contact with lake water"	G-I symptoms: 24-48 hours		N/S	Affected individuals all holidaying in rental cottages. Well water to cottages free of bacterial contamination	[68]
1979	Late summer	Arrowhead Lake, PA, USA	Single female	N/S	N/S ("young girl")	Swimming #	Chills, sore throat, fever, nausea, diarrhoea	N/S	"within several hours"	Approx. 3 days		N/S		[68]
1989	Early autumn	Rudyard Lake, Staffs, UK	2	N/S	16	Swimming and canoeing exercises, the latter involving 360^0 rolls * #	Malaise, sore throat, circum-oral blistering, left-sided pleuritic pain, dry cough, vomiting, central abdominal pain, diarrhoea, fever	*M. aeruginosa*	Day after exposure	<2 weeks	Microcystin-LR found to be principal cyanobacterial toxin present	N/S	Both were hospitalised, having developed left lower-lobe pneumonia accompanied by thrombocytopenia	[23, 57, 74, 160, 161]
1989	Early autumn	Rudyard Lake, Staffs, UK	8	N/S	N/S (soldiers)	As above * #	Sore throat, headache, abdominal pain, dry cough, diarrhoea, vomiting, blistered mouth	*M. aeruginosa*	N/S	N/S	See above	N/S	All subjects were soldiers engaged in canoeing exercises, subsequently admitted to barracks medical centre	[23, 57, 74, 160, 161]

Year	Season	Location	Number affected	Estimates of number unaffected	Age	Water activity	Signs & symptoms	Dominant plankton	Time of onset after exposure	Symptom duration	Diagnostic criteria	Predisposing conditions	Notes	References
**Cold & flu-like symptoms (incl. fever, headache, myalgia, arthralgia, sore throat, respiratory and gastro-intestinal)**														
1992	Mid-spring	Darling River, Wilcannia, NSW, Australia	2	N/S	N/S - teenagers	Swimming	Gastroenteritis and myalgia	*Anabaena* sp	N/S	Symptoms resolved after 48 hrs	One subject required admission to hospital.	N/S	Some town residents reported itchy skin rashes after showering, even after town water supply was carbon-filtered	[162]
N/S	N/S	Unnamed reservoir, UK	Single subject, male	N/S	39	Windsurfing	Fever, nausea, vomiting	N/S, but microcystin isolated from reservoir; several sheep and dogs died	N/S "shortly after windsurfing trip"	N/S	Liver function tests, liver biopsy	No significant past history, no medications	Mild hepatic dysfunction investigated six weeks after exposure	[163]
**Mixed symptoms**														
1945	Late summer	Lake Keesus, Waukesha County, WI, USA	Report of single subject, female	N/S	39	Swimming	Gross eyelid oedema, nasal congestion, generalised urticarial rash	N/S	"while swimming"	N/S	Skin test with Oscillatoreaceae extract (0.03mL of 1:10,000 dilution) resulted in immediate and severe local reaction treated by local injection of adrenaline	Long-standing history of autumn hay-fever and seasonal asthma	Oscillatoreaceae extracts elicited positive (but unspecified) reactions in "many individuals who knew that swimming caused hay fever" at dilutions up to 1:100,000	[53]
1946	Late summer	Lake Keesus, WI, USA	Same subject	N/S		Swimming	As above	N/S	N/S	N/S				[53]
1973-4	Summer	Belgrano Park pond, Santa Fe City, Argentina	N/S	N/S	N/S	Swimming and bathing	G-I symptoms, dermatitis, otitis, conjunctivitis	Mainly *M. aeruginosa*; bloom contained "up to 60,000 colonies/mL"	N/S	N/S	N/S	N/S		[164]
1979	Late summer	Arrowhead Lake, PA, USA	Initial reports: 20-30 children, several adults. 12 children + I adult investigated	N/S	4-12, + adult	Swimming	Headache: 8/13 stomach cramps: 9/13 nausea: 5/13 vomiting: 7/13 diarrhoea: 11/13 fever: 5/13 rash: 1/13 sore or inflamed throat: 3/13	N/S	During exposure, to 12 hours after exposure. Rash on arms & legs of adult ♀ developed "shortly after wading along lake edge"	Most symptoms: <72 hours All symptoms: ≤5 days	Stool samples of four children negative for *Salmonella* and *Shigella*. Throat swab of one child with sore throat negative for viral involvement	N/S	Routine weekly monitoring for faecal coliforms showed counts were ≤40/100mL prior to incident	[68]

Year	Season	Location	Number affected	Estimates of number unaffected	Age	Water activity	Signs & symptoms	Dominant plankton	Time of onset after exposure	Symptom duration	Diagnostic criteria	Predisposing conditions	Notes	References
**Mixed symptoms**														
1980	Mid-summer	Pocono Highlands Lake, PA, USA	N/S "swimmers"	N/S	N/S	Swimming	Eye irritation, earache, sore throat	*Anabaena* sp.	N/S	N/S	Water samples produced signs of poisoning in mice suggestive of hepatotoxin. LD_{50} = 90 mg/kg (i.p. mouse)	N/S		[56]
1980	Late summer	Lake Lahonton, NV, USA	N/S "several… affected "	N/S	N/S	Water skiing, swimming	Erythema, eye irritation, dizziness, nausea, stomach cramps, diarrhoea	*Aphanizomenon flos-aquae*	N/S	N/S	LD_{50} = 500 mg/kg body weight (i.p. mouse)	N/S		[56]
1980	Mid-summer	Camp William Penn, PA, USA	75-100	N/S	N/S	N/S ("campers affected from contact with water")	Conjunctivitis, sore-red throat, headaches, diarrhoea, nausea	*Anabaena* sp.	N/S	N/S		N/S		[56]
1981	Mid-summer	Harveys Lake, PA, USA	N/S ("many reports…")	N/S	N/S	N/S	Skin irritation, nausea, dizziness, diarrhoea	*Anabaena flos-aquae*	N/S	N/S	Mouse toxicity testing of water: neurotoxins and hepatotoxins present. LD_{50} = 125 mg/kg	N/S	Lake closed to public access until early spring	[56]
1989	Early autumn	Rutland Water, Leics, UK, and other UK lakes	N/S ("several")	N/S	N/S	Sail-boarding #	Skin rashes, nausea, vomiting, blistering inside mouth, severe thirst	*M. aeruginosa*	N/S	N/S	Microcystin-LR found to be principal cyanobacterial toxin present	N/S	20 sheep and 15 dogs died over approx. 3 weeks in late summer – early spring after contact with bloom scum	[6, 65, 160]
1990	Spring	Lake Cargelligo, NSW, Australia	2 or 3	N/S	N/S	Swimming	Two cases of conjunctivitis, one case of rash	*Anabaena circinalis*	N/S	N/S		N/S	Lake closed as water supply for one month while bloom evident	[66]
1991	Summer-Autumn	Lakes Alexandrina and Albert, SA, Australia	1	N/S	N/S (adult)	Crayfishing	Skin and/or eye symptoms (pruritus, skin rash, sore red eyes)	*Nodularia* sp	N/S	N/S	Case identified retrospectively by either interview with local residents, local health workers or surveillance through local GPs	N/S	Seven more cases identified with skin and/or eye symptoms. Two of them also had asthma symptoms, one had hay fever symptoms, another a sore throat. Water contact was by showering or bathing	[165]

Year	Season	Location	Number affected	Estimates of number unaffected	Age	Water activity	Signs & symptoms	Dominant plankton	Time of onset after exposure	Symptom duration	Diagnostic criteria	Predisposing conditions	Notes	References
**Mixed symptoms**														
1991	Late spring – early summer	Darling River, Wilcannia, NSW, Australia	1	Sole report after surveillance requests to report confirmed or suspected cyanobacteria -related illness during an extensive riverine bloom	N/S (adult)	Water skiing	Skin rash, conjunctivitis, diarrhoea, respiratory difficulty	*Anabaena* spp	N/S	N/S		N/S	All schools along the river system were asked to report increases in illness-related absenteeism, especially for G-I symptoms. No such increase was reported	[157]
1991-2	Summer	River Murray, SA, Australia	11	N/S	1-64	“water sport…. particularly skiing”. One of these cases had skin contact through both water sport and residential water use	Contact exposure (n=2): rash, itching, mouth blistering, eye irritation; oral ingestion (n=3): diarrhoea, vomiting, nausea, muscle weakness, sore throat, respiratory difficulty, headache; contact + oral exposure (n=6): mixture of above symptoms	Predominantly *A. circinalis*	N/S	N/S	Cases investigated following telephone or personal complaints to health authorities and water supply management	N/S	15 further cases with exposure to River Murray water from reticulated water supply	[166]
1994	N/S	Lake Velencei, Hungary	100-150	N/S	Children at a youth camp	N/S	skin and eye irritation	“heavy bloom” of *M. aeruginosa*	N/S	N/S	N/S	N/S		[167]
1996	N/S	Hollingworth Lake, UK	11	N/S	N/S (sea cadets)	Canoe capsizing trials *	Facial rashes, asthmatic signs, dry sporadic cough, vomiting	*Planktothrix agardhii*, containing three microcystins	Day of, and after exposure	N/S		N/S		[23]
1997	Autumn	Lake Sammamish, WA, USA	N/S (“several”)	N/S	“young children”	Swimming	G-I complaints, rashes	*M. aeruginosa*, microcystins measured at approx. 500 µg/g dry weight	N/S	N/S	N/S	N/S	A dog began “heaving and coughing”, died four hours after exposure to bloom	[45, 46]

Year	Season	Location	Number affected	Estimates of number unaffected	Age	Water activity	Signs & symptoms	Dominant plankton	Time of onset after exposure	Symptom duration	Diagnostic criteria	Predisposing conditions	Notes	References
**Mixed symptoms**														
2003	N/S	Lake Iso-Kukkanen, Finland	1	N/S	2 yo child	Swimming	Fever and eye irritation	*A. lemmermannii* sole or dominant cyanobacterium.	N/S	N/S		N/S	Saxitoxin measured at 13-270 µg/L (depending on methods). Low levels of microcystins found. Anatoxin-a and degradation products reported	[158]

# – cyanobacteria-affected water reportedly ingested (table column: “Water activity”)

* – occupational exposure (table column: “Water activity”)

N/S – not stated
